# Perfusion deficits may underlie lung and kidney injury in severe COVID-19 disease: insights from a multicenter international cohort study

**DOI:** 10.1186/s44158-024-00175-1

**Published:** 2024-07-06

**Authors:** Alice Nova, Bairbre McNicholas, Aurora Magliocca, Matthew Laffey, Vanessa Zambelli, Ilaria Mariani, Minahel Atif, Matteo Giacomini, Giovanni Vitale, Roberto Rona, Giuseppe Foti, John Laffey, Emanuele Rezoagli, The LUNG-KIDNEY Interaction Multicenter Study Group, Aine O’Connor, Marco Giani, Matteo Pozzi, Andrea Coppadoro, Silvia Sordi, Ilaria Alice Crippa

**Affiliations:** 1 School of Medicine and Surgery, University of Milano-Bicocca, Monza, Italy; 2 School of Medicine, National University of Ireland Galway, Galway, Ireland; 3 Department of Anesthesia and Intensive Care Medicine, Galway University Hospitals, Galway, Ireland; 4 Department of Anesthesia and Intensive Care Medicine, Gruppo Ospedaliero San Donato, Policlinico San Marco, Zingonia, Bergamo, Italy; 5 Department of Pathophysiology and Transplants, University of Milan, Milan, Italy; 6 School of Medicine, Trinity College Dublin, Dublin 2, Ireland; 7 Department of Emergency and Intensive Care, Fondazione IRCCS San Gerardo dei Tintori Hospital, Monza, Italy

**Keywords:** Respiratory failure, Acute kidney injury, Dead space, Perfusion, Mechanical ventilation, Minute ventilation

## Abstract

**Background:**

Lung perfusion defects, mainly due to endothelial and coagulation activation, are a key contributor to COVID-19 respiratory failure. COVID-19 patients may also develop acute kidney injury (AKI) because of renal perfusion deficit. We aimed to explore AKI-associated factors and the independent prediction of standardized minute ventilation (MV)—a proxy of alveolar dead space—on AKI onset and persistence in COVID-19 mechanically ventilated patients.

**Methods:**

This is a multicenter observational cohort study. We enrolled 157 COVID-19 patients requiring mechanical ventilation and intensive care unit (ICU) admission. We collected clinical information, ventilation, and laboratory data. AKI was defined by the 2012 KDIGO guidelines and classified as transient or persistent according to serum creatinine criteria persistence within 48 h. Ordered univariate and multivariate logistic regression analyses were employed to identify variables associated with AKI onset and persistence.

**Results:**

Among 157 COVID-19 patients on mechanical ventilation, 47% developed AKI: 10% had transient AKI, and 37% had persistent AKI. The degree of hypoxia was not associated with differences in AKI severity. Across increasing severity of AKI groups, despite similar levels of paCO_2_, we observed an increased MV and standardized MV, a robust proxy of alveolar dead space. After adjusting for other clinical and laboratory covariates, standardized MV remained an independent predictor of AKI development and persistence. d-dimer levels were higher in patients with persistent AKI.

**Conclusions:**

In critically ill COVID-19 patients with respiratory failure, increased wasted ventilation is independently associated with a greater risk of persistent AKI. These hypothesis-generating findings may suggest that perfusion derangements may link the pathophysiology of both wasted ventilation and acute kidney injury in our population.

**Supplementary Information:**

The online version contains supplementary material available at 10.1186/s44158-024-00175-1.

## Background

Kidney and lung injuries are common and associated with significant morbidity and mortality in critically ill patients [1, 2]. Patients with acute kidney injury (AKI) are more likely to require mechanical ventilation [1, 2], whereas an increased risk for AKI was observed in patients with acute respiratory failure or acute respiratory distress syndrome (ARDS) [3]. The co-existence of AKI, even mild or moderate, with ARDS was associated with prolonged ICU and hospital stay, prolonged duration of mechanical ventilation, and higher hospital mortality in a secondary analysis [4] of the LUNG SAFE study [5]. The recent COVID-19 pandemic sparked once again the interest about the lung—kidney crosstalk in critically ill patients. Despite initial reports [6], it has been observed that AKI frequency reaches 30% among patients with COVID-19 requiring ICU admission and mechanical ventilation [7, 8].

Several mechanisms explain how lung injury may adversely affect kidney function in the context of “typical” ARDS. First, the reduced cardiac output due to positive pressure ventilation [9, 10] and/or venous stasis [11] may reduce renal blood flow. In the COVID-19 ARDS, the transmission of the pulmonary pressures to the thoracic compartment and its effect on the renal perfusion may be even more pronounced [12, 13], since respiratory system mechanics are often preserved, when compared with typical ARDS [14]. Second, the changes in the partial pressure of oxygen and carbon dioxide may affect renal perfusion by influencing vascular resistance [15]. Finally, in the context of ventilatory-induced lung injury (VILI), biotrauma may lead to systemic inflammation and organ dysfunction via the release of inflammatory cytokines [16, 17]. When considering the interplay between COVID-19-related respiratory and kidney failure, unique features of SARS-CoV-2 infection have to be considered. The SARS-CoV-2 virus interaction with the angiotensin‐converting enzyme 2 (ACE2) receptor, widely expressed on the renal epithelium, could lead to direct damage to the renal parenchyma [18]. Kidney injury could be also systemically mediated by inflammatory cell recruitment, macrophage activation syndrome, and cytokine storm [19]. The systemic inflammation promotes the activation of deranged coagulation pathways, leading to perfusion impairment in renal small vessels. Autopsy studies supported the concept of renal “malperfusion,” reporting glomerular and peritubular capillaries thrombosis associated with glomerular ischemia [20, 21]. In COVID-19 respiratory failure, the presence of perfusion deficits in systemic and pulmonary circulation has been reported [22, 23]. Additionally, coagulopathy has been identified as an independent predictor for pulmonary and systemic thromboembolic events [22]. These findings may suggest a disease-specific AKI characterized by thromboembolic manifestations, coagulation impairment, and endothelial cell swelling with foamy degeneration resulting in endothelialitis [21, 22]. Interestingly, in COVID-19 respiratory failure, the activation of deranged coagulation, as assessed by increasing levels of d-dimer, was associated with a significant alveolar dead space, resulting in an increased respiratory workload [23].

Whether the presence of dead space in COVID-19 respiratory failure—as result of coagulation activation and ventilation-perfusion mismatch—may explain kidney malperfusion and the development of AKI is yet to be demonstrated. We hypothesize that acute kidney and lung injury may be driven by common and linked pathophysiologic processes including coagulation activation and perfusion deficit.

In this multicenter retrospective study, we aimed to investigate whether laboratory parameters suggestive of inflammation and coagulation activation, along with ventilatory indices indicating the severity of lung injury and increased ventilatory load are associated with AKI development and persistence [24].

## Methods

### Study design and data collection

In this multi-center international retrospective observational study, we enrolled 161 consecutive adult patients, with a diagnosis by real-time PCR of COVID-19 pneumonia requiring mechanical ventilation and ICU admission from February to May 2020. Exclusion criteria were age less than 18 years old and pregnancy. The participating centers included IRCSS Fondazione San Gerardo dei Tintori (Monza, Italy), Policlinico San Marco (Zingonia – Bergamo, Italy), and Galway University Hospitals (Galway, Ireland). This research was part of the STORM Study approved by Istituto Nazionale Malattie Infettive Lazzaro Spallanzani, Rome, Italy (Resolution no. 84/2020; NCT04424992). The local ethics committee of Galway University Hospitals, Galway (C.A. 2384), and Policlinico San Marco, Zingonia (Reg. Sperim. N. 118/20), approved the study. Informed consent was waived considering the observational, non-interventional nature of the study.

Demographic, anamnestic, and clinical data of all patients were collected in a dedicated database. Laboratory data, including complete blood count, coagulation panel, liver and kidney function tests, and arterial blood gas analysis, were daily recorded from the day of ICU admission to day 7 and then every 7 days until day 28. Ventilatory settings and ventilatory mechanics parameters were recorded in the same way. The need for adjunctive therapies such as neuromuscular blockade, pronation, inhaled nitric oxide, veno-venous extracorporeal membrane oxygenation (V-V ECMO), and renal replacement therapy (RRT) was recorded. Furthermore, we collected data about ICU outcomes.

We defined and staged AKI by using serum creatinine over 7 days from ICU admission, according to the 2012 Kidney Disease Improving Global Outcomes (KDIGO) clinical practice guidelines [25]. In most of the clinical charts, the hourly urinary output was either unavailable or unreliable (e.g., not precisely recorded every hour) due to the pandemic context. For these reasons, we decided to base our AKI definition and classification solely on the creatinine parameter, consistent with literature from the pandemic period [26, 27].

Baseline renal function was defined using serum creatinine within six months prior to ICU admission when available. Otherwise, we estimated serum creation by using the following formula, as reported as 2021 CKD—EPI creatinine [28].

$$\text{Serum creatinine }= 142\text{ x }{(\text{Scr}/\text{A})}^{\text{B}}\text{ x }{0.9938}^{\text{age}}\text{ x }(1.012\text{ if female})$$

where A and B are 0.7 and − 1.2, respectively, if female and 0.9 and − 1.2 if male. Persistent AKI was defined by the continuance of AKI by serum creatinine criteria, according to the KDIGO guidelines, beyond 48 h from its onset. If a complete and sustained reversal of the AKI episode occurred within 48 h, we categorized the AKI as transient [24]. Patients with chronic kidney disease (CKD) at any stage were excluded from statistical analysis, considering that serum creatine estimate is not reliable in this population and that one of the most significant risk factors for AKI is pre-existing CKD and AKI itself plays a role in both the CKD development and the progression of pre-existing CKD [29].

### Statistical analysis

Normality was assessed using the Shapiro-Wilk test, and continuous data are expressed as mean ± SD or median [IQR], as appropriate. Categorical data are expressed as count (proportion). The test for the trend of normally distributed variables across ordered groups was performed by using linear regression, while the trend of non-normally distributed variables was tested by Jonckheere-Terpstra test. Differences in continuous data were assessed by unpaired Student’s *T* test or *U* Mann-Whitney test, as appropriate. Categorical data across ordered groups were evaluated via Cochran-Armitage trend test. Chi-square test was performed to compare categorical data between two groups. The effect of the AKI group and the day from the ICU admission on continuous variables was tested using two-way ANOVA. Post hoc analysis was conducted using the Sidak correction method to evaluate the difference in the continuous variable between different AKI groups. The correlations between increasing AKI stages (i.e., No AKI, transient AKI, persistent AKI) and qualitative and quantitative variables were obtained using ordered univariable and multivariable logistic regressions. The factors associated with a more severe AKI stage by using a univariable ordered logistic regression analysis (*p* < 0.05) and considered clinically meaningful were included in ordered logistic multivariable models. The significance level was set to 5% (two-sided). Stata/MP version 17 (Copyright 1985–2021 StataCorp LLC, College Station, TX, USA), and IBM SPSS Statistics (IBM SPSS Statistics for Macintosh, Version 20.0. Armonk, NY: IBM Corp) were used for the statistical analyses.

## Results

### Study population

We enrolled 161 patients admitted to the intensive care units of the participating centers from February to May 2020. Eighty-nine (55%) patients were admitted to Monza, 51 (32%) at Zingonia, and 21 (13%) at Galway Hospital. We excluded four patients with chronic kidney disease. Thus, 157 patients were included in the final analysis. All patients presented hypoxemic respiratory failure due to COVID-19 requiring ICU admission and invasive mechanical ventilation. We stratified the population into three groups based on the temporary presence and persistence of AKI during the ICU stay. Seventy-three (46.5%) patients developed AKI. Of these, 10 (15%) showed a transient AKI, whereas in 58 (37%) patients, the AKI was persistent. We observed more patients without AKI in Monza hospital, while a higher incidence of persistent AKI was reported in Zingonia hospital. No differences in demographic characteristics and main comorbidities were observed among the three groups (Table 1).

**Table 1:** Baseline characteristics and renal characterization of the study population

	**No AKI, *n* = 84** **(53%)**	**Transient AKI, *n* = 15** **(10%)**	**Persistent AKI, *n* = 58** **(37%)**	*P* value for trend*
**Center**				
San Gerardo, Monza, n (%)	60 (71)	8 (53)	20 (34)	< 0.001
	60 (68)	8 (9)	20 (23)	
Policlinico San Marco, Zingonia, n (%)	16 (19)	4 (27)	30 (52)	< 0.001
	16 (32)	4 (8)	30 (60)	
University Hospital, Galway, n (%)	8 (10)	3 (20)	8 (14)	0.411
	8 (42)	3 (16)	8 (42)	
**Demographics**				
Age (years)	61 [55–68]	65 [59–69]	62 [56–67]	0.566
BMI (kg/m^2^)	28 [25–31]	29 [26–33]	29 [26–32]	0.273
Male, n (%)	65 (77)	10 (67)	44(76)	0.797
**Comorbidities**				
COPD, *n* (%)	3 (4)	0 (0)	3(5)	0.659
Asthma, *n* (%)	3 (4)	1 (7)	4 (7)	0.368
Chronic heart failure, *n* (%)	2 (2)	0 (0)	1 (2)	0.754
Cancer (solid or hematologic), *n* (%)	2 (2)	1 (7)	3 (5)	0.375
Diabetes, *n* (%)	14 (17)	2 (13)	9 (15)	0.841
Systemic hypertension, *n* (%)	32 (38)	10 (67)	27 (46)	0.270
Atrial fibrillation, *n* (%)	2 (2)	0 (0)	2 (3)	0.708
OSAS, *n* (%)	1 (1)	1 (7)	0(0)	0.611
**Renal characteristics**				
Baseline creatinine (mg/dL)	1.09 [1.03–1.24]	1.07 [0.84–1.12]	1.09 [1.04–1.13]	0.518
Creatinine at ICU admission (mg/dL)	0.80 [0.70–0.90]	1.15 [0.93–1.50]	1.10 [0.80–1.56]	< 0.001
Creatinine peak within 7 days (mg/dL)	0.90 [0.80–1.15]	1.20 [1.00–1.60]	1.75 [1.00–3.90]	< 0.001
Urea at ICU admission (mg/dL)	39 [28–45]	53 [34–88]	56 [32–68]	< 0.001
First 24 h urinary output (mL/kg/h)	0.58 [0.38–0.94]	0.77 [0.56–0.90]	0.50 [0.32–0.88]	0.530
First 24 h fluid balance (mL)	490 [–121–1114]	660 [375–1399]	916 [5–1640]	0.031
Cumulative fluid balance (mL)	1133 ± 3010	630 ± 3751	2160 ± 3457	0.131
CRRT (%)	0 (0)	2 (13)	16 (28)	< 0.001
Diuretics, *n* (%)	83 (99)	13 (87)	50 (89)	0.015
Nephrotoxic drugs, *n* (%)^a^	30 (36)	4 (27)	8 (14)	0.004
KDIGO AKI stage, *n* (%)				
Stage 1	/	5 (33)	2 (3)	< 0.001
Stage 2	/	3 (20)	7 (12)	0.426
Stage 3	/	7 (47)	49 (84)	0.002

Continuous data will be expressed as mean ± SD or median [IQR], as appropriate; categorical variables were reported as count (proportion) (*n*, %)

Definition of abbreviation. *BMI* Body mass index, *COPD* Chronic obstructive pulmonary disease, *CRRT* Continuous renal replacement therapy, *ICU* Intensive care unit, *OSAS* Obstructive sleep apnea syndrome

^*^P values for trend were calculated with the use of linear regression, Jonckheere-Terpstra, and Cochran-Armitage trend test, where appropriate

^a^Nephrotoxic drugs included non-steroidal anti-inflammatory drugs (NSAIDs), Colistin, Aminoglycosides, and Vancomycin

### Renal characteristics

**Fig. 1:**
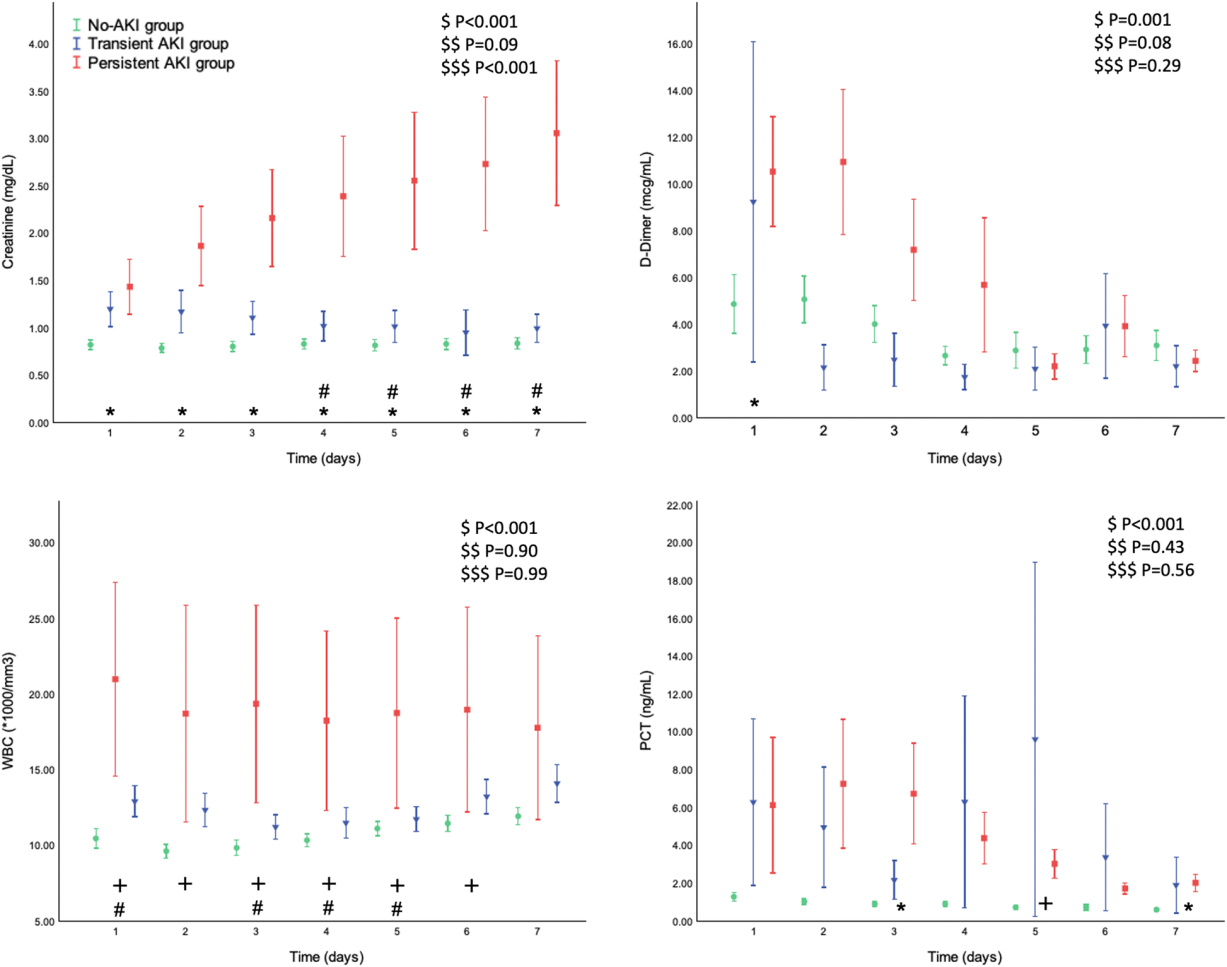
Creatinine, d-dimer, WBC, and PCT during the first ICU week, stratified in no AKI, transient AKI, and persistent AKI groups. The trend in creatinine, d-dimer, WBC, and PCT over time in patients without AKI (green circles), with transient (blue triangles), and persistent (red squares) AKI. All data represent mean ± SEM. ^$^ refers to the effect of AKI groups on the continuous variable; ^$$^ refers to the effect of day from ICU admission on the continuous variable; ^$$$^ refers to the interaction between AKI groups and day from ICU admission on the continuous variable. ^✚^*P* < 0.05 for comparison between no AKI and transient AKI groups; *P < 0.05 for comparison between no-AKI and persistent AKI groups; ^**#**^*P* < 0.05 for comparison between transient and persistent AKI groups. Definition of abbreviation. *PCT*, procalcitonin; *WBC*, white blood cells

Baseline creatinine was similar among the three groups. As compared with baseline, a trend toward higher levels of creatinine and urea at ICU admission and higher creatinine peak within 7 days in patients with persistent AKI was observed (Table 1). The levels of creatinine remained higher in the persistent AKI group during the first 7 days after ICU admission (Fig. 1). A higher fluid balance in the first 24 h was observed in the persistent AKI group. Among the patients who developed transient and persistent AKI, 2 (13%) and 16 (28%) patients required continuous renal replacement therapy (CRRT), respectively (Table 1).

### Laboratory data at ICU admission

To investigate a possible role of inflammation in the development of AKI, we analyzed inflammatory markers and coagulation function among the three patients’ groups. White blood cells were higher in patients who developed AKI compared to those who did not, while inflammatory markers and coagulation function were similar among the three groups (Table 2). During the first 7 days of ICU, patients who developed AKI exhibited higher levels of inflammatory markers and a trend toward higher values of white blood cells. A significant effect of AKI groups on d-dimer level and a trend toward more elevated d-dimer level was observed in the persistent AKI patients (Fig. 1). A more severe metabolic impairment was observed in patients with AKI (Table 2).

**Table 2:** Laboratory, ventilatory, and hemodynamic parameters during ICU stay

	**No AKI, *n* = 84** **(53%)**	**Transient AKI, *n* = 15** **(10%)**	**Persistent AKI, *n* = 58** **(37%)**	*P* value for trend*
**Laboratory parameters at ICU admission**				
CRP (mg/dL)	19.42 [9.05–24.27]	18.54 [12.20–28.05]	23.20 [11.10–28.30]	0.163
PCT (ng/mL)	0.56 [0.30–1.73]	0.75 [0.28–6.34]	1.06 [0.29–3.94]	0.187
WBC (*10^3^/mm^3^)	9.34 [6.98–12.16]	12.70 [11.70–20.16]	11.05 [8.77–14.40]	0.009
Platelets (*10^3^/mm^3^)	256 [187–339]	273 [170–334]	225 [190–305]	0.306
INR	1.15 [1.09–1.28]	1.11 [1.07–1.16]	1.14 [1.05–1.32]	0.728
aPTT ratio	0.98 [0.88–1.06]	0.88 [0.79–1.00]	0.92 [0.85–1.00]	0.072
Fibrinogen (mg/dL)	639 [525–768]	677 [567–788]	609 [365–750]	0.521
Dimer (µg/mL)	1.02 [0.50–3.01]	1.36 [0.57–4.33]	1.58 [0.54–22]	0.388
LDH (IU/L)	474 [404–583]	598 [340–792]	488 [392–632]	0.919
Bilirubin (mg/dL)	0.60 [0.40–0.80]	0.80 [0.40–1.40]	0.70 [0.44–1.12]	0.062
Albumin (mg/dL)	2.90 [2.50–3.10]	3.10 [2.80–3.20]	2.90 [2.70–3.30]	0.159
pH	7.37 ± 0.82	7.33 ± 0.07	7.32 ± 0.11	0.002
Bicarbonate (mmol/L)	26 [23–28]	24 [21–25]	24 [21–27]	0.020
Base Excess (mmol/L)	0.80 [–1–05–3.00]	–0.05 [–1.00–1.40]	–0.2 [–3.6–2.7]	0.017
Lactate (mmol/l)	1.5 [1.2–2–0]	2.0 [1.4–2.3]	1.8 [1.3–2.7]	0.009
Sodium (mEq/L)	140 [137–142]	138 [136–142]	140 [138–143]	0.041
Potassium (mEq/L)	3.9 [3.5–4.2]	4.0 [3.8–4.3]	4.0 [3.5–4.5]	0.088
Chloride (mEq/L)	103 [101–106]	104 [101–105]	104 [101–108]	0.264
Strong ion difference (mEq/L)	40 [38–41]	39 [38–41]	41 [39–44]	0.047
**Severity of critical illness at ICU admission**				
Cpl, rs (ml/cmH_2_O)	41 [34–45]	39 [32–43]	40 [31–48]	0.817
DP (cmH_2_O)	11 [9–13]	12.5 [10–14]	11 [10–14]	0.116
PaO_2_/ FiO_2_	151 [121–187]	190 [128–208]	139 [100–168]	0.161
PaCO_2_ (mmHg)	45 [39–53]	42 [33–61]	48 [39–65]	0.193
EtCO_2_ (mmHg)	39 [34–41]	40 [37–45]	35 [33–38]	0.037
Dead space (%)	37 [32–38]	37 [26–44]	39 [34–41]	0.827
MV (L/min)	10 [8–11]	11 [8–12]	11 [9–12]	0.044
Standardized MV (L/min)	11 [9–13]	12 [10–13]	13 [10–19]	0.022
SOFA score	6 [4–8]	8 [4–9]	9 [6–13]	< 0.001
Cardiovascular SOFA score	0 [0–3]	3 [0–3]	3 [0–4]	0.005
NMBDs, *n* (%)	78 (93)	12 (80)	51 (89)	0.451
	71 (84)	11 (73)	40 (69)	0.027
iNO, *n* (%)	6 (7)	0 (0)	2 (3)	0.310
V–V ECMO, *n* (%)	5 (6)	1 (7)	0(0)	0.075
**Ventilatory parameters—average of the first ICU week**				
PEEP^c^ (cmH_2_O)	13 [12–15]	12 [11–14]	13 [12–14]	0.004
TV^c^ (mL)	440 [393–460]	420 [380–437]	449 [410–490]	0.036
TV/IBW^c^ (mL/kg)	6 [6, 7]	7 [5.5–7.5]	6 [6, 7]	0.503
RR^c^ (breaths/min)	24 [22–26]	24 [23–28]	26 [21–28]	0.173
MV^c^ (L/min)	10 [9–12]	10 [8–11]	11 [9–14]	0.024
Standardized MV^ac^ (L/min)	12 [10–16]	12 [10–14]	13 [10–18]	0.073
Plateau pressure^c^ (cmH_2_O)	24 [22–26.5]	24 [21–26.5]	25 [22–28]	0.339
Cpl, rs^c^ (ml/cmH_2_O)	39.5 [34–45]	39 [31.5–43]	40 [31.5–48]	0.935
DP^c^ (cmH_2_O)	11 [9–12]	10.5 [9.5–12.5]	12 [10–13.5]	0.141
EtCO_2_^c^ (mmHg)	39 [34–41]	40 [37–45]	35 [33–38]	0.038
PaO_2_^c^ (mmHg)	90 [82–97]	86 [75–107]	85 [75–93]	0.037
PaCO_2_^c^ (mmHg)	50 [46–54]	50 [42–54]	49 [43–60]	0.960
FiO_2_^c^ (%)	61 [51–72]	55 [46–70]	70 [54–83]	0.038
PaO_2_/ FiO_2_^c^	151 [121–187]	190 [128–208]	139 [100–168]	0.161
Dead space^bc^ (%)	23 [18–32]	18 [10–31]	31 [24–43]	0.049
**Hemodynamic parameters—average of the first ICU week**				
MAP^c^ (mmHg)	80 ± 7	83 ± 9	81 ± 8	0.497
CVP^c^ (mmHg)	11 ± 3	12 ± 2	10 ± 3	0.362
HR^c^ (beats/min)	87 ± 13	89 ± 12	94 ± 15	0.007
Lactate^c^ (mmol/l)	1.5 [1.2–1.8]	1.6 [0.9–2.2]	1.7 [1.4–2.4]	0.012
**Outcomes**				
Bacterial over-infection, *n* (%)	48 (57)	7 (47)	18 (31)	0.002
Stroke, *n* (%)	0 (0)	0 (0)	2 (3)	0.076
VTE, *n* (%)	24 (29)	5 (33)	9 (15)	0.083
PTE, *n* (%)	10 (12)	0 (0)	6 (10)	0.704
IMV duration (days)	14 [10–24]	11 [6–37]	6 [3–12]	< 0.001
NIMV duration (days)	1 [0–2]	1 [0–2]	0 [0–1]	< 0.001
Tracheostomy, *n* (%)	20 (24)	3 (20)	5 (9)	0.019
VFDs (days)	12 [0–17]	0 [0–21]	0 [0–22]	0.134
ICU LOS (days)	15 [11–27]	12 [8–37]	6 [2–17]	< 0.001
Hospital LOS (days)	36 [24–50]	31 [14–52]	14 [7–34]	< 0.001
ICU mortality, *n* (%)	14 (17)	3 (20)	29 (50)	< 0.001
Renal recovery, *n* (%)	/	8 (53)	19 (33)	0.141†

Continuous data will be expressed as mean ± SD or median [IQR], as appropriate; categorical variables were reported as count (proportion) (*n*, %)

Definition of abbreviation. *ALT* Alanine transaminase, *aPTT* Activated partial thromboplastin time, *AST aspartate* Aminotransferase, *CRP* C-reactive protein, *CVP* Central venous pressure, *Cpl*,*rs* Compliance of respiratory system, *DP* Driving pressure, *EtCO*_*2*_ End-tidal CO_2_, *FiO*_*2*_ Inspiratory fraction of oxygen, *HR* Heart rate, *IBW* Ideal body weight, *ICU LOS* Intensive care unit length of stay, *IMV* Invasive mechanical ventilation, *iNO* Inhaled nitric oxide, *INR* International normalized ratio, *LDH* Lactate dehydrogenase, *MAP* Mean arterial pressure, *MV* Minute ventilation, *NIMV* Non-invasive mechanical ventilation, *PaCO*_*2*_ Arterial partial pressure of carbon dioxide, *PaO*_*2*_ Arterial partial pressure of oxygen, *PCT* Procalcitonin, *PEEP* Positive end-expiratory pressure, *PTE* Pulmonary thromboembolism, *RR* Respiratory rate, *SOFA* Sequential organ failure assessment, *TV* Tidal volume, *VFDs* Ventilator-free days, *VTE* Venous thromboembolism, *V-V ECMO* Veno-venous extracorporeal membrane oxygenation, *WBC* White blood cells

^*^P values for trend were calculated with the use of linear regression, Jonckheere-Terpstra, and Cochran-Armitage trend test, where appropriate

^†^Chi^2^ test was used to estimate differences in the proportions of renal recovery between transient and persistent AKI groups

^a^Standardized minute ventilation = minute ventilation × PaCO_2_/40 mm Hg

^b^Dead space = (PaCO_2_- EtCO_2_/PaCO_2_)*100

^c^Refers to the average of the first 7 days of ICU stay

### Respiratory parameters

Upon admission, the PaO_2_/FiO_2_ ratio was similar in patients who developed persistent or transient AKI and in patients who did not. The median of PaO_2_ during the first 7 ICU days was lower in AKI patients. Patients were ventilated with higher median PEEP and FiO_2_ levels in the first week of ICU stay by moving through AKI classes. The respiratory system mechanics at ICU admission were similar among the groups (Table 2). No difference was observed in arterial CO_2_ tension at ICU admission, but a significant trend toward a lower-end tidal CO_2_ on admission and during the first week was reported in AKI groups (Table 2). Furthermore, the median dead space, calculated as the difference between PaCO_2_ and end-tidal CO_2_ divided by PaCO_2_ [30], increased significantly across the three cohorts during the first ICU week (Table 2).

**Fig. 2:**
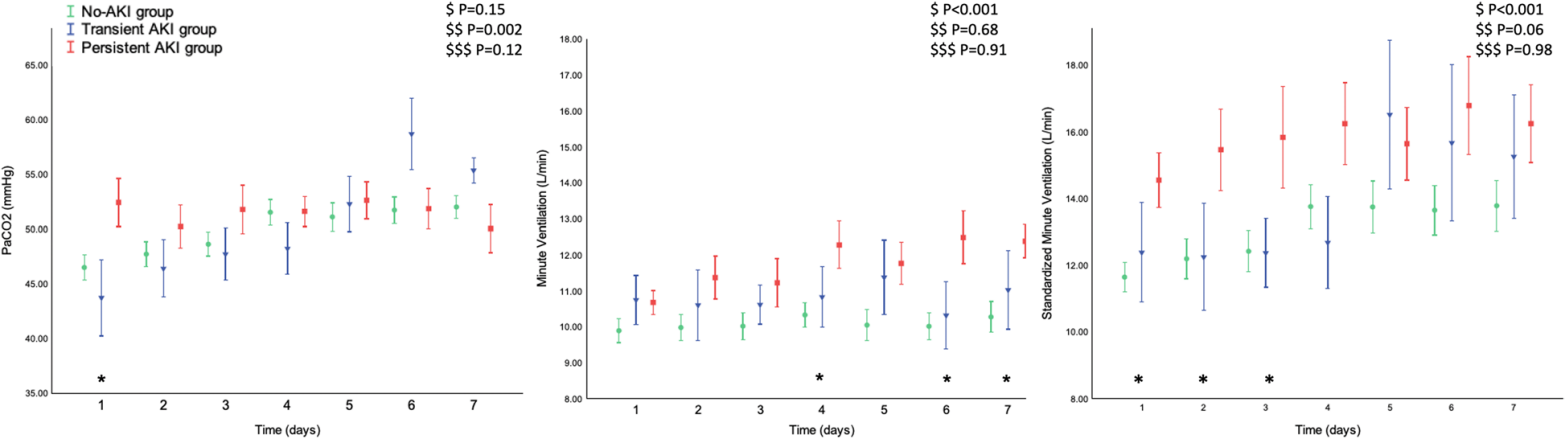
PaCO_2_, minute ventilation, and standardized minute ventilation, during the first ICU week, stratified in no AKI, transient AKI, and persistent AKI groups. The trend in PaCO_2_, minute ventilation, and standardized minute ventilation over time in patients without AKI (green circles), with transient (blue triangles), and persistent (red squares) AKI. All data represent mean ± SEM. ^$^ refers to the effect of AKI groups on the continuous variable; ^$$^ refers to the effect of day from ICU admission on the continuous variable; ^$$$^ refers to the interaction between AKI groups and day from ICU admission on the continuous variable. ^✚^*P* < 0.05 for comparison between no-AKI and transient AKI groups; *P < 0.05 for comparison between no-AKI and persistent AKI groups; ^**#**^*P* < 0.05 for comparison between transient and persistent AKI groups. Definition of abbreviation. *PaCO*_*2*_, arterial partial pressure of carbon dioxide

On admission and during the first week of ICU, patients presented a higher minute ventilation from no AKI to higher severity classes of AKI (Fig. 2). The proportion of patients who underwent prone positioning decreased from the no-AKI to the persistent AKI group, while no difference in the use of neuromuscular blocking drugs, inhaled nitric oxide and V-V ECMO was observed in the two groups (Table 2). The overall population of patients who underwent prone position was ventilated with higher PEEP levels than those who did not. Patients who underwent prone position in no-AKI and persistent AKI groups at ICU admission (Additional file 1: Table S1)—as well as during the first ICU week (Additional file 1: Table S2)**—**were exposed to higher PEEP levels than those who did not.

**Fig. 3:**
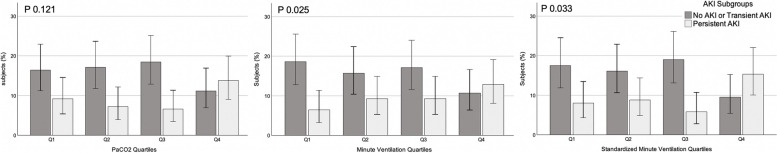
Subjects (%) stratified in no AKI/transient AKI and persistent AKI subgroups in ordinal groups of PaCO_2_, minute ventilation, and standardized minute ventilation. PaCO_2_ (mmHg): Q1 < 38.7, Q2 ≥ 38.7 - < 45.1, Q3 ≥ 45.1 - < 56.2, Q4 ≥ 56.2; minute ventilation (L/min): Q1 < 8.7, Q2 ≥ 8.7 - < 10.3, Q3 ≥ 10.3 - < 12.0, Q4 ≥ 12.0; standardized minute ventilation (L/min): Q1 < 9.6, Q2 ≥ 9.6 - < 11.8, Q3 ≥ 11.8 - < 15.3, Q4 ≥ 15.3. All data represent the percentage of subjects in the overall population (%); *P* values represent Cochran-Armitage trend tests and error bars represent 95% confidence intervals

**Fig. 4:**
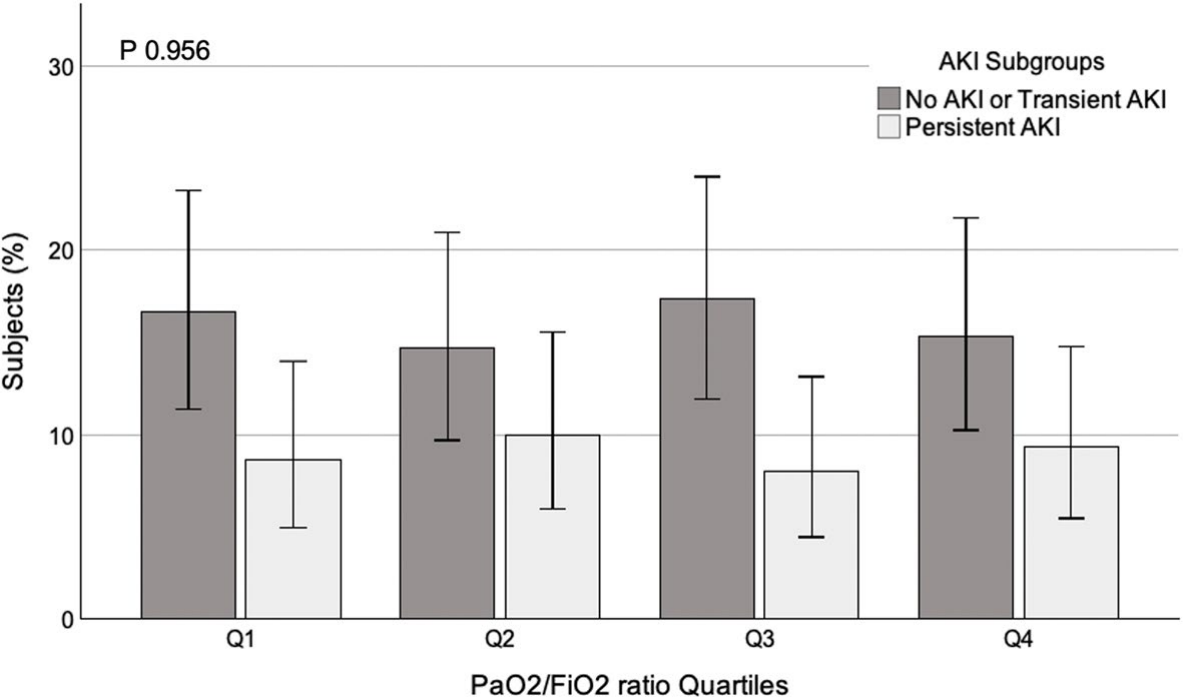
Subjects (%) stratified in no AKI/transient AKI and persistent AKI subgroups in ordinal groups of PaO_2_/FiO_2_ ratio. PaO_2_/FiO_2_ ratio: Q1 < 85, Q2 ≥ 85 - < 114, Q3 ≥ 114 - < 157, Q4 ≥ 157. All data represent the percentage of subjects in the overall population (%). *P* values represent Cochran-Armitage trend tests, and error bars represent 95% confidence intervals

Upon dividing AKI subgroups into ordinal quartiles of respiratory load indicators, we noted a significantly higher percentage of subjects developing persistent AKI in the higher MV and standardized MV quartiles and a trend toward a higher percentage of persistent AKI in the quartiles with higher PaCO_2_ (Fig. 3). We did not observe any difference in the percentage of persistent AKI patients among the PaO_2_/FiO_2_ ratio quartiles (Fig. 4).

To assess whether standardized MV [5], as an index of the ventilatory load needed to keep PaCO_2_ within the normal range, may independently affect AKI incidence and persistence in severe COVID-19 patients, we performed an ordered multivariable analysis. After adjusting for confounders, including non-respiratory SOFA score, bicarbonate level at ICU admission, prone position, and admission center, standardized MV remained independently associated with a higher risk to develop AKI and AKI persistence (Table 3).

**Table 3:** Ordered multivariable analysis of factors independently associated with the probability of development and persistence of AKI

**Variable**	**OR (95% CI)**	**(95% CI)**	***P* value**
Non-respiratory SOFA score (unit increase)	1.30	1.11–1.52	0.001
Bicarbonate (1 mMol/L)	0.91	0.83–1.01	0.065
Prone position (Ref. No)	0.45	0.15–1.38	0.165
Center (Ref. Zingonia)			
• Monza	1.24	0.38–4.04	0.719
• Galway	0.66	0.11–3.99	0.650
Standardized MV^a^	1.10	1.01–1.21	0.036

*N* = 134

Definition of abbreviation: *MV* Minute ventilation, *SOFA* Sequential organ failure assessment

^a^Standardized minute ventilation = minute ventilation × PaCO_2_/40 mm Hg

### Hemodynamic parameters

We examined mean arterial pressure, heart rate, central venous pressure, vasopressor requirement, and lactate levels during the first ICU week in our population to evaluate the relationship between hemodynamics and renal function. A trend toward a more severe hemodynamic impairment was observed in patients with AKI. Patients with persistent AKI presented higher lactates, heart rate, and cardiovascular SOFA scores. No difference was reported in mean arterial pressure and central venous pressure among the groups (Table 2).

### Outcomes

A similar incidence of thromboembolic complications (i.e., stroke, venous thromboembolism (VTE), pulmonary thromboembolism (PTE)) among the groups was found. Patients without AKI show a higher rate of bacterial over-infection during their ICU stay. Patients with persistent AKI show shorter duration of both invasive and non-invasive ventilation and shorter ICU and hospital length of stay. A significant tendency to higher ICU mortality was observed by moving through the AKI spectrum (Table 2). No difference in renal recovery was described when comparing transient AKI and persistent AKI classes (Table 2).

## Discussion

The main results of this study can be summarized as follows:

- Among 157 COVID-19 patients on mechanical ventilation, 47% developed AKI: 10% had transient AKI, and 37% had persistent AKI.
- Across increasing severity of AKI groups, despite similar levels of paCO_2_, we observed an increased minute ventilation and a higher standardized minute ventilation, a robust proxy of dead space.
- The degree of hypoxia was not associated with differences in AKI severity.
- After adjusting for other clinical and laboratory covariates, standardized minute ventilation remained an independent predictor of AKI development and persistence.
- d-dimer levels were increased early after ICU admission only in the persistent AKI group.

Among mechanically ventilated COVID-19 patients admitted to the ICU, AKI is a common yet severe complication. In our cohort, we observed a 46.5% incidence of AKI. Similar results were found in a recent study testing AKI incidence in patients with severe COVID-19, they observed a 53% AKI incidence in their patients [7]. In severe COVID-19 patients, a higher AKI incidence than in “typical” ARDS has been reported [3, 4]. Independent risk factors for AKI in non-COVID-19 ARDS include sepsis, non-cardiogenic shock, transfusion-related acute lung injury, and pancreatitis [31, 32], which were absent or extremely rare in our population. Therefore, other factors must be examined as potential causes of AKI in our cohort. We acknowledge that in emergency settings, such as during the initial European wave of COVID-19, the impracticality of maintaining a high standard of care may lead to severe respiratory distress, dehydration, and hypovolemia, potentially resulting in more severe kidney failure. Patients with persistent AKI showed to have higher serum creatinine and urea at ICU admission and a more positive fluid balance during the first 24 h, underling the severity of the organ failure in this group. Furthermore, in the persistent AKI group, we observed a higher percentage of patients with KDIGO stage 3 AKI, which has been associated with an increased risk of mortality in a COVID-19-related ARDS [29]. The rapid shortage of ICU beds was particularly important in small centers [6]. The surge of patients admitted to the hospital and the lack of ICU beds may be the reason for a higher incidence of AKI in Zingonia Hospital, a peripheric center very close to the Italian pandemic epicenter when compared with Monza and Galway hospitals.

We decided to stratify our patients according to the ADQI definition of transient and persistent AKI [24], considering the clinical relevance of this recent classification. When considering our cohort of AKI patients, 10 (21%) subjects developed transient AKI, while 58 (79%) had persistent AKI. In a retrospective analysis of all-cause ICU patients, the incidence of transient and persistent AKI was 73.4 and 16.5%, respectively [33]. Conversely, in septic patients, the incidence of transient and persistent AKI was 18.4% and 81.6%, and sustained AKI was found to be independently associated with sepsis mortality, as well as inflammatory and procoagulant responses [34]. In our study, COVID-19-related AKI in ICU was frequent and persistent. This finding is consistent with previous literature [35] and may suggest some overlapping mechanisms with sepsis-related AKI. We observed a significant trend toward higher ICU mortality in the persistent AKI group, in line with a previous report, where persistent AKI has been correlated with higher progression to CKD, morbidity, and mortality [36].

When analyzing inflammatory biomarkers, white blood cell count at ICU admission showed a higher trend in persistent AKI patients at ICU admission and during the first 3 days of ICU. Despite similar serum procalcitonin (PCT) at ICU admission, higher PCT levels were found during the first ICU week. Prolonged persistence of plasma PCT should include both the possibility of a sustained production and an impaired renal clearance of procalcitonin in the context of AKI [37]. Unexpectedly, a lower rate of bacterial over-infection was observed in the two AKI groups compared with the patients without AKI, suggesting that the sustained inflammation and its possible role in AKI persistence may not be due to bacterial over-infection.

A sustained inflammatory response and consequent coagulation activation may play a role in the development and persistence of COVID-19-related AKI. The SARS-Cov2 virus causes a dysregulation of the immune response leading to cytokine storm and macrophage activation syndrome. In this context, the complement activation stimulates coagulation pathways [38]. Inflammation and coagulation stimulate each other and may expose the kidney to intensive and repeated stimuli. Furthermore, glomerular and peritubular capillaries obstruction, due to erythrocyte aggregation and fibrin thrombi, has suggested a role of kidney thrombotic microangiopathy in COVID-19-related AKI pathophysiology [20, 21]. The significant effect of the AKI group on d-dimer, along with a higher level of d-dimer observed in the persistent AKI group when compared with the no AKI group on the first day of ICU may support this pathological mechanism in our population.

Despite no difference in the severity of hypoxemic respiratory failure (i.e., similar PaO_2_/FiO_2_ ratio and respiratory system mechanics at ICU admission) among the subgroups, an increased need for higher MV at ICU admission was required through the AKI spectrum. Furthermore, a higher MV was also required in the persistent AKI cohort during the following days until day 7. When considering the average respiratory parameters during the first week of ICU, we observed a significant trend towards a lower EtCO_2_ and a higher estimated dead space among the three groups. We noted a significantly higher percentage of subjects developing persistent AKI in the higher MV and standardized MV quartiles with a trend toward a higher percentage of persistent AKI in the quartiles with more hypercapnic patients. Sustained hypercapnia was not associated with an increase in serum creatinine in non-COVID-19 ARDS [39]. However, our findings are consistent with previous reports about critically ill COVID-19 and ARDS patients, where an increased dead space [40, 41] and a significant impairment in CO_2_ clearance [39] were described.

We were not able to support the perfusion deficit as the leading cause of increased dead space through autopsy evaluations or imaging techniques. However, previous autopsy data reported inflammatory infiltration of endothelial cells and micro-thrombosis in the lung tissue of COVID-19 patients [42].

In our cohort, standardized MV is independently associated with the onset and persistence of AKI after adjusting for confounding factors. Although standardized MV does not directly measure alveolar dead space, it can be considered a strong proxy of dead space [30], as it effectively characterizes the additional ventilatory load required to maintain PaCO_2_ within the normal range. An increase in standardized MV has been observed with the severity of ARDS [5].

The absence of association between bicarbonate with AKI in the multivariable model suggests that the increase in the MV is probably aimed at keeping acceptable CO_2_ levels because of higher dead space in the presence of AKI. Even if our hypothesis needs to be further investigated from a histologic and imaging point of view, our preliminary findings suggest that a perfusion deficit may be a common mechanism in the development of lung and kidney failure.

The proportion of patients who underwent prone positioning decreased from the no-AKI to the persistent AKI group. Furthermore, patients who underwent prone positioning were ventilated with higher PEEP levels, than those who did not, which may be explained by a higher severity of respiratory failure in patients undergoing prone position. The benefits related to a more homogeneous ventilation and distribution of total stress and strain, which leads to a reduced risk of VILI [43, 44], may have a protective role against AKI development and persistence by improving lung compliance and gas exchange and reducing the systemic effect of biotrauma [16, 17]. This finding underscores the crucial role of personalized PEEP titration [45] and positional therapy in ARDS [46, 47], as well as the need to combine lung and kidney protection. Recently, Fogagnolo and colleagues proposed the Renal Resistive Index (RRI) as a promising non-invasive diagnostic tool to predict the risk of AKI in mechanically ventilated patients and that the PEEP setting may contribute to modulate the resistance of the renal blood flow and be associated with the onset of AKI [48].

We observed a trend toward more severe hemodynamic impairment in patients with persistent AKI. We can suppose that despite the attempt to ensure an adequate cardiac output through a compensatory increase of heart rate and a higher dose of vasoactive/inotropic drugs (i.e., similar mean arterial pressure among the group but higher cardiovascular SOFA score in AKI patients), the peripheral perfusion was suboptimal in patients who developed sustained AKI. Therefore, we cannot exclude that a reduction of renal blood flow may be a contributing factor to AKI development.

This study has some limitations. First, we based the AKI definition only on serum creatinine over 7 days from ICU admission, according to the 2012 KDIGO cutoff [25], because the hourly urinary output was not available on the clinical charts. This may lead to an underestimation of the AKI cohort in our population [49]. Furthermore, given the well-known limitations of serum creatinine in the assessment and prognostication of AKI, new biomarkers of kidney injury may offer further insights into characterizing COVID-19-associated AKI [50]. Secondly, we estimated serum creatinine for the patients in which the baseline renal function was not available within six months prior to the ICU admission. Even if the 2021 CKD—EPI creatinine formula [28] has been demonstrated to be accurate, some misclassifications may occur. Finally, no histological findings were available in our research to prove the potential common mechanism of increased dead space and kidney injury from a biological standpoint.

## Conclusions

In conclusion, in a population of critically ill patients with COVID-19 respiratory failure requiring ICU admission and mechanical ventilation, a high rate of wasted ventilation and dead space is linked to a higher risk of developing persistent AKI. Our finding generated the hypothesis that acute kidney and lung injury may be driven by common and chained pathophysiologic processes within a lung-kidney injury cross-talk. The concept of alveolar dead space, as ventilated but non-perfused parenchyma, in the presence of significant systemic inflammatory activation, may be applied to other organs. In this scenario, various pathophysiologic mechanisms in COVID-19-related ARDS—such as vascular thromboembolism and vasoconstriction—could impair renal perfusion and filtration, resulting in a shared scenario of lung and “renal dead space”.

### Supplementary Information

Additional file 1: Table S1. PEEP at ICU admission in patients who underwent prone position and who did not, stratified by AKI groups. Table S2. PEEP (average during the first ICU week) in patients who underwent prone position and who did not, stratified by AKI groups.

## Data Availability

The datasets used and/or analyzed during the current study are available from the corresponding author on reasonable request.

## Abbreviations

AKI: Acute kidney injury
ALT: Alanine transaminase
aPTT: Activated partial thromboplastin time
ARDS: Acute respiratory distress syndrome
AST aspartate: Aminotransferase
BMI: Body mass index
CKD: Chronic kidney disease
COPD: Chronic obstructive pulmonary disease
CRP: C-reactive protein
CRRT: Continuous renal replacement therapy
CVP: Central venous pressure
Cpl,rs: Compliance of respiratory system
DP: Driving pressure
EtCO_2_: End-tidal CO_2_
FiO_2_: Inspiratory fraction of oxygen
HR: Heart rate
IBW: Ideal body weight
ICU: Intensive care unit
ICU LOS: Intensive care unit length of stay
IMV: Invasive mechanical ventilation
iNO: Inhaled nitric oxide
INR: International normalized ratio
KDIGO: Kidney Disease Improving Global Outcomes
LDH: Lactate dehydrogenase
MAP: Mean arterial pressure
MV: Minute ventilation
NIMV: Non-invasive mechanical ventilation
OSAS: Obstructive sleep apnea syndrome
PaCO_2_: Arterial partial pressure of carbon dioxide
PaO_2_: Arterial partial pressure of oxygen
PCT: Procalcitonin
PEEP: Positive end-expiratory pressure
PTE: Pulmonary thromboembolism
RR: Respiratory rate
RRT: Renal replacement therapy
SOFA: Sequential organ failure assessment
TV: Tidal volume
VFDs: Ventilator-free days
VTE: Venous thromboembolism
V-V ECMO: Veno-venous extracorporeal membrane oxygenation
WBC: White blood cells

## References

[1] Hoste EA, Bagshaw SM, Bellomo R (2015). Epidemiology of acute kidney injury in critically ill patients: the multinational AKI-EPI study. Intensive Care Med.

[2] Ostermann M, Chang RW (2011). Impact of different types of organ failure on outcome in intensive care unit patients with acute kidney injury. J Crit Care.

[3] Darmon M, Clec'h C, Adrie C (2014). Acute respiratory distress syndrome and risk of AKI among critically ill patients. Clin J Am Soc Nephrol.

[4] McNicholas BA, Rezoagli E, Pham T (2019). Impact of early acute kidney injury on management and outcome in patients with acute respiratory distress syndrome: a secondary analysis of a multicenter observational study. Crit Care Med.

[5] Bellani G, Laffey JG, Pham T (2016). Epidemiology, patterns of care, and mortality for patients with acute respiratory distress syndrome in intensive care units in 50 countries [published correction appears in JAMA. 2016 Jul 19;316(3):350] [published correction appears in JAMA. 2016 Jul 19;316(3):350]. JAMA.

[6] Grasselli G, Pesenti A, Cecconi M (2020). Critical care utilization for the COVID-19 outbreak in Lombardy, Italy: early experience and forecast during an emergency response. JAMA.

[7] Fabrizi F, Alfieri CM, Cerutti R, Lunghi G, Messa P (2020). COVID-19 and acute kidney injury: a systematic review and meta-analysis. Pathogens.

[8] Yang X, Yu Y, Xu J (2020). Clinical course and outcomes of critically ill patients with SARS-CoV-2 pneumonia in Wuhan, China: a single-centered, retrospective, observational study [published correction appears in Lancet Respir Med. 2020 Apr;8(4):e26]. Lancet Respir Med.

[9] Murdaugh HV, Sieker HO, Manfredi F (1959). Effect of altered intrathoracic pressure on renal hemodynamics, electrolyte excretion and water clearance. J Clin Invest.

[10] Hall SV, Johnson EE, Hedley-Whyte J (1974). Renal hemodynamics and function with continuous positive-pressure ventilation in dogs. Anesthesiology.

[11] Pesenti A, Slobod D, Magder S (2023). The forgotten relevance of central venous pressure monitoring. Intensive Care Med.

[12] Fogagnolo A, Grasso S, Dres M (2022). Focus on renal blood flow in mechanically ventilated patients with SARS-CoV-2: a prospective pilot study. J Clin Monit Comput.

[13] Ottolina D, Zazzeron L, Trevisi L (2022). Acute kidney injury (AKI) in patients with COVID-19 infection is associated with ventilatory management with elevated positive end-expiratory pressure (PEEP). J Nephrol.

[14] Gattinoni L, Coppola S, Cressoni M, Busana M, Rossi S, Chiumello D (2020). COVID-19 does not lead to a “typical” acute respiratory distress syndrome. Am J Respir Crit Care Med.

[15] Darmon M, Schortgen F, Leon R (2009). Impact of mild hypoxemia on renal function and renal resistive index during mechanical ventilation. Intensive Care Med.

[16] Liu KD, Glidden DV, Eisner MD (2007). Predictive and pathogenetic value of plasma biomarkers for acute kidney injury in patients with acute lung injury. Crit Care Med.

[17] Brower RG, Matthay MA, Acute Respiratory Distress Syndrome Network (2000). Ventilation with lower tidal volumes as compared with traditional tidal volumes for acute lung injury and the acute respiratory distress syndrome. N Engl J Med.

[18] Hamming I, Timens W, Bulthuis ML, Lely AT, Navis G, van Goor H (2004). Tissue distribution of ACE2 protein, the functional receptor for SARS coronavirus. A first step in understanding SARS pathogenesis. J Pathol.

[19] Zaim S, Chong JH, Sankaranarayanan V, Harky A (2020). COVID-19 and multiorgan response. Curr Probl Cardiol.

[20] Duarte-Neto AN, Monteiro RAA, da Silva LFF (2020). Pulmonary and systemic involvement in COVID-19 patients assessed with ultrasound-guided minimally invasive autopsy. Histopathology.

[21] Su H, Yang M, Wan C (2020). Renal histopathological analysis of 26 postmortem findings of patients with COVID-19 in China. Kidney Int.

[22] Klok FA, Kruip MJHA, van der Meer NJM (2020). Incidence of thrombotic complications in critically ill ICU patients with COVID-19. Thromb Res.

[23] Grasselli G, Tonetti T, Protti A (2020). Pathophysiology of COVID-19-associated acute respiratory distress syndrome: a multicentre prospective observational study. Lancet Respir Med.

[24] Chawla LS, Bellomo R, Bihorac A (2017). Acute kidney disease and renal recovery: consensus report of the acute disease quality initiative (ADQI) 16 Workgroup. Nat Rev Nephrol.

[25] Kidney Disease: Improving Global Outcomes (KDIGO) Acute Kidney Injury Work Group (2012). KDIGO clinical practice guideline for acute kidney injury. Kidney Int Suppl.

[26] Hirsch JS, Ng JH, Ross DW (2020). Acute kidney injury in patients hospitalized with COVID-19. Kidney Int.

[27] Gupta S, Hayek SS, Wang W (2020). Factors associated with death in critically ill patients with coronavirus disease 2019 in the US [published correction appears in JAMA Intern Med. 2020 Nov 1;180(11):1555] [published correction appears in JAMA Intern Med. 2021 Aug 1;181(8):1144]. JAMA Intern Med.

[28] Inker LA, Eneanya ND, Coresh J (2021). New creatinine- and cystatin C-based equations to estimate GFR without race. N Engl J Med.

[29] Rezoagli E, McNicholas BA, Madotto F (2022). Presence of comorbidities alters management and worsens outcome of patients with acute respiratory distress syndrome: insights from the LUNG SAFE study. Ann Intensive Care.

[30] Rezoagli E, Laffey JG, Bellani G (2022). Monitoring lung injury severity and ventilation intensity during mechanical ventilation. Semin Respir Crit Care Med.

[31] Rezoagli E, McNicholas B, Pham T, Bellani G, Laffey JG (2020). Lung-kidney cross-talk in the critically ill: insights from the Lung Safe study. Intensive Care Med.

[32] McNicholas BA, Rezoagli E, Simpkin AJ, et al (2023) Correction : Epidemiology and outcomes of early-onset AKI in COVID-19-related ARDS in comparison with non-COVID-19-related ARDS: insights from two prospective global cohort studies. Crit Care 27(1):202. 10.1186/s13054-023-04487-6. Published 2023 May 26 10.1186/s13054-022-04294-5 PMC9814373 36604753

[33] Mo S, Bjelland TW, Nilsen TIL, Klepstad P (2022). Acute kidney injury in intensive care patients: Incidence, time course, and risk factors. Acta Anaesthesiol Scand.

[34] Uhel F, Peters-Sengers H, Falahi F (2020). Mortality and host response aberrations associated with transient and persistent acute kidney injury in critically ill patients with sepsis: a prospective cohort study. Intensive Care Med.

[35] Rubin S, Orieux A, Prevel R (2020). Characterization of acute kidney injury in critically ill patients with severe coronavirus disease 2019. Clin Kidney J.

[36] Kellum JA, Sileanu FE, Bihorac A, Hoste EA, Chawla LS (2017). Recovery after acute kidney injury. Am J Respir Crit Care Med.

[37] Meisner M, Lohs T, Huettemann E, Schmidt J, Hueller M, Reinhart K (2001). The plasma elimination rate and urinary secretion of procalcitonin in patients with normal and impaired renal function. Eur J Anaesthesiol.

[38] Tuculeanu G, Barbu EC, Lazar M (2023). Coagulation disorders in sepsis and COVID-19-two sides of the same coin? A review of inflammation-coagulation crosstalk in bacterial sepsis and COVID-19. J Clin Med.

[39] Madotto F, Rezoagli E, McNicholas BA (2020). Patterns and impact of arterial CO_2_ management in patients with acute respiratory distress syndrome: insights from the LUNG SAFE study. Chest.

[40] Graf J, Pérez R, López R (2022). Increased respiratory dead space could associate with coagulation activation and poor outcomes in COVID-19 ARDS. J Crit Care.

[41] Vasques F, Sanderson B, Formenti F, Shankar-Hari M, Camporota L (2020). Physiological dead space ventilation, disease severity and outcome in ventilated patients with hypoxaemic respiratory failure due to coronavirus disease 2019. Intensive Care Med.

[42] Varga Z, Flammer AJ, Steiger P (2020). Endothelial cell infection and endotheliitis in COVID-19. Lancet.

[43] Xin Y, Cereda M, Hamedani H (2018). Unstable inflation causing injury. Insight from prone position and paired computed tomography scans. Am J Respir Crit Care Med.

[44] Motta-Ribeiro GC, Hashimoto S, Winkler T (2018). Deterioration of regional lung strain and inflammation during early lung injury. Am J Respir Crit Care Med.

[45] Rezoagli E, Bellani G (2019). How I set up positive end-expiratory pressure: evidence- and physiology-based!. Crit Care.

[46] Rezoagli E, Bastia L, Brochard L, Bellani G (2023). Physical manoeuvres in patients with ARDS and low compliance: bedside approaches to detect lung hyperinflation and optimise mechanical ventilation. Eur Respir J.

[47] Dougal A, Rezoagli E, Pham T (2020). Patterns of use of adjunctive therapies in patients with early moderate to severe ARDS: insights from the LUNG SAFE study. Chest.

[48] Fogagnolo A, Grasso S, Morelli E et al (2024) Impact of positive end-expiratory pressure on renal resistive index in mechanical ventilated patients. J Clin Monit Comput. 10.1007/s10877-024-01172-z. Published online May 21 10.1007/s10877-024-01172-z PMC11427533 38771490

[49] Montomoli J, Rezoagli E, Bellini V, Finazzi S, Bignami EG (2023) A "generalized wayfinding" paradigm for improving AKI understanding and classification: insights from the Dutch registries. Minerva Anestesiol 89(7-8):724–726. 10.23736/S0375-9393.23.17256-7. in the manuscript flow 10.23736/S0375-9393.23.17256-7 36943712

[50] Yoon SY, Kim JS, Jeong KH, Kim SK (2022). Acute kidney injury: biomarker-guided diagnosis and management. Medicina (Kaunas).

