# Supplementary material for: Perfusion deficits may underlie lung and kidney injury in severe COVID-19 disease: insights from a multicenter international cohort study
Source: J Anesth Analg Crit Care. 2024 Jul 6;4:40. doi: 10.1186/s44158-024-00175-1 (PMC11227201; doi:10.1186/s44158-024-00175-1)
Supplement: Supplementary file 1 — Additional file 1: Table S1. PEEP at ICU admission in patients who underwent prone position and who did not, stratified by AKI groups. Table S2. PEEP (average during the first ICU week) in patients who underwent prone position and who did not, stratified by AKI groups. [file 44158_2024_175_MOESM1_ESM.docx]

**Supplemental material**

**Perfusion deficits may underlie lung and kidney injury in severe COVID-19 disease: Insights from a multicenter international cohort study**

**Authors:**

Nova Alice^1*^, McNicholas Bairbre^2,3*^, Magliocca Aurora^4,5^, Laffey Matthew^6^, Zambelli Vanessa^1^, Mariani Ilaria^1^, Minahel Atif^2,3^, Giacomini Matteo^4^, Vitale Giovanni^4^, Roberto Rona^7^, Foti Giuseppe^1,7^, Laffey John^2,3#^, Rezoagli Emanuele^1,7#^

**Study collaborators:**

Aine O’Connor, from the Department of Anesthesia and Intensive Care Medicine, Galway University Hospitals; Giacomo Bellani, Marco Giani and Matteo Pozzi, from the School of Medicine and Surgery, University of Milano-Bicocca, Monza, Italy and Department of Emergency and Intensive Care, Fondazione IRCCS San Gerardo dei Tintori, Monza, Italy; Silvia Sordi and Ilaria Alice Crippa, from the Department of Anesthesia and Intensive Care Medicine, Policlinico San Marco, Gruppo Ospedaliero San Donato, Zingonia, Bergamo, Italy.

**Affiliations:**

^1^School of Medicine and Surgery, University of Milano-Bicocca, Monza, Italy

^2^School of Medicine, University of Galway, Galway, Ireland

^3^Department of Anesthesia and Intensive Care Medicine, Galway University Hospitals

^4^Department of Anesthesia and Intensive Care Medicine, Policlinico San Marco, Gruppo Ospedaliero San Donato, Zingonia, Bergamo, Italy

^5^Department of Medical Physiopathology and Transplants, University of Milan, Milano, Italy

^6^School of Medicine, Trinity College Dublin, Dublin 2, Ireland

^7^Department of Emergency and Intensive Care, Fondazione IRCCS San Gerardo dei Tintori, Monza, Italy

*share co-first authorship

^#^share co-senior authorship

**Corresponding author:**

Emanuele Rezoagli, MD, PhD

School of Medicine and Surgery, University of Milano-Bicocca, Monza, Italy

Department of Emergency and Intensive Care, Fondazione IRCCS San Gerardo dei Tintori, Monza, Italy

Tel.: +390392339273; Email: [emanuele.rezoagli@unimib.it](mailto:emanuele.rezoagli@unimib.it)

**Table 1. PEEP at ICU admission in patients who underwent prone position and who did not, stratified by AKI groups.**

| **PEEP (cmH_2_O)** | **No Prone Position** | **Prone Position** | **P-value** |
| --- | --- | --- | --- |
| **Overall** | 11 [10-12] | 14 [12-15] | <0.001 |
| **No-AKI** | 12 [10-13] | 14 [13-15] | <0.001 |
| **Transient AKI** | 11 [10-12] | 12 [12-16] | 0.145 |
| **Persistent AKI** | 10.5 [10-12] | 15 [12-15] | <0.002 |

Data are reported as median [interquartile range]. AKI=acute kidney injury; PEEP=positive end-expiratory pressure.

**Table 2. PEEP (average during the first ICU week) in patients who underwent prone position and who did not, stratified by AKI groups.**

| **PEEP (cmH_2_O)** | **No Prone Position** | **Prone Position** | **P-value** |
| --- | --- | --- | --- |
| **Overall** | 10 [10-12] | 14 [13-15] | <0.001 |
| **No-AKI** | 10 [10-12] | 14 [13-15] | <0.001 |
| **Transient AKI** | 10.5 [10-11] | 13 [11-14] | 0.131 |
| **Persistent AKI** | 11 [10-12] | 13 [12-14] | <0.001 |

Data are reported as median [interquartile range]. AKI=acute kidney injury; PEEP=positive end-expiratory pressure.
